# Mechanisms of Membrane Curvature Generation in Membrane Traffic

**DOI:** 10.3390/membranes2010118

**Published:** 2012-02-29

**Authors:** Hye-Won Shin, Hiroyuki Takatsu, Kazuhisa Nakayama

**Affiliations:** 1 Career-Path Promotion Unit for Young Life Scientists, Kyoto University, Sakyo-ku, Kyoto 606-8501, Japan; E-Mail: takachu@cp.kyoto-u.ac.jp; 2 Graduate School of Pharmaceutical Sciences, Kyoto University, Sakyo-ku, Kyoto 606-8501, Japan; E-Mail: kazunaka@pharm.kyoto-u.ac.jp

**Keywords:** membrane trafficking, small GTPase, BAR domain, P4-ATPase, membrane curvature

## Abstract

During the vesicular trafficking process, cellular membranes undergo dynamic morphological changes, in particular at the vesicle generation and fusion steps. Changes in membrane shape are regulated by small GTPases, coat proteins and other accessory proteins, such as BAR domain-containing proteins. In addition, membrane deformation entails changes in the lipid composition as well as asymmetric distribution of lipids over the two leaflets of the membrane bilayer. Given that P4-ATPases, which catalyze unidirectional flipping of lipid molecules from the exoplasmic to the cytoplasmic leaflets of the bilayer, are crucial for the trafficking of proteins in the secretory and endocytic pathways, changes in the lipid composition are involved in the vesicular trafficking process. Membrane remodeling is under complex regulation that involves the composition and distribution of lipids as well as assembly of proteins.

## 1. Introduction

Biological membranes not only separate the interior of the cell from the external environment, but also bring about compartmentalization of distinct subcellular organelles. Intracellular compartments along the secretory and endocytic pathways communicate with one another through membrane trafficking, specifically that mediated by carrier vesicles. Membrane trafficking ensures delivery of appropriate materials, such as proteins and lipids, in order to maintain the identity of these organelles. Dynamic membrane shape changes occur during formation of the vesicles and tubules that mediate cargo delivery in the secretory and endocytic pathways. These carrier intermediates vary in shape, from small vesicles (~50 nm) and thin tubules to tubular-saccular carriers [1,2,3]. 

Upon membrane deformation, a selected membrane subdomain is curved, sensed and stabilized by a variety of proteins. Formation of carrier intermediates is driven through a complex process that involves modification and changes in composition and distribution of lipids, as well as local assembly of coat protein complexes and proteins involved in sensing and forming membrane curvature. In particular, insertion of amphipathic helices into the membrane subdomain is required for curvature generation; proteins containing BAR (Bin/Amphiphysin/Rvs) domains, which form a crescent-like homodimer, are required for sensing and stabilizing this curvature. Here, we will introduce the idea of local curvature generation via changes in the inter-leaflet distribution of lipids, catalyzed by P4-ATPases, and insertion of the amphipathic helices of Arf family small GTPases. Furthermore, we will discuss how the curvature is stabilized by BAR domain proteins. We also consider the interplays among these proteins that generate and stabilize membrane curvature in the context of membrane trafficking. 

## 2. Lipids

### 2.1. Relationship between Asymmetric Lipid Distribution and Membrane Deformation

Lipid bilayers, including those of the plasma membrane and the membrane-enclosed compartments along the secretory and endocytic pathways, exhibit asymmetric lipid distributions. In particular, aminophospholipids, phosphatidylserine (PS) and phosphatidylethanolamine (PE) are enriched in the cytoplasmic leaflet. For example, in resting human red blood cells, PS and PE are restricted primarily to the inner/cytoplasmic leaflet of the plasma membrane, whereas phosphatidylcholine (PC) and sphingomyelin (SM) are exposed on the cell surface [4,5]. Regulated exposure of PS in the outer/exoplasmic leaflet occurs during a variety of biological processes, such as apoptotic cell death, platelet coagulation and myotube formation [5,6,7,8]. On the other hand, PE is exposed on the surface of the cleavage furrow during cytokinesis [9]. 

Drastic cell shape changes occur upon exogenous application of lyso-PC or impermeable amphipathic drugs. The former flips into the inner leaflet of the bilayer at a slow rate; the latter intercalate only into the exterior leaflet of the bilayer; these phenomena can be explained by the bilayer couple mechanism [10,11]. Moreover, insertion of lyso-PC into a giant unilamellar vesicle induces formation of a single bud [12]. These observations suggest that changes in distribution of specific lipids between the two leaflets of bilayers are able to drive membrane deformation. 

### 2.2. Phospholipid Flippases (P4-ATPases)

An ATP-dependent “flippase” (also called aminophospholipid translocase) activity in the plasma membrane of human erythrocytes was discovered by Seigneuret and Devaux [13]. Subsequently, P4-ATPases, a subfamily of P-type ATPases, were recognized as the most likely candidates for flippases in eukaryotic membranes [14,15,16]. P4-ATPases have been implicated in flipping aminophospholipids from the exoplasmic/lumenal leaflet to the cytoplasmic leaflet of cellular membranes [14,17,18,19,20]. In yeast, it has also been reported that even PC can be translocated by P4-ATPases [18].

**Figure 1:**
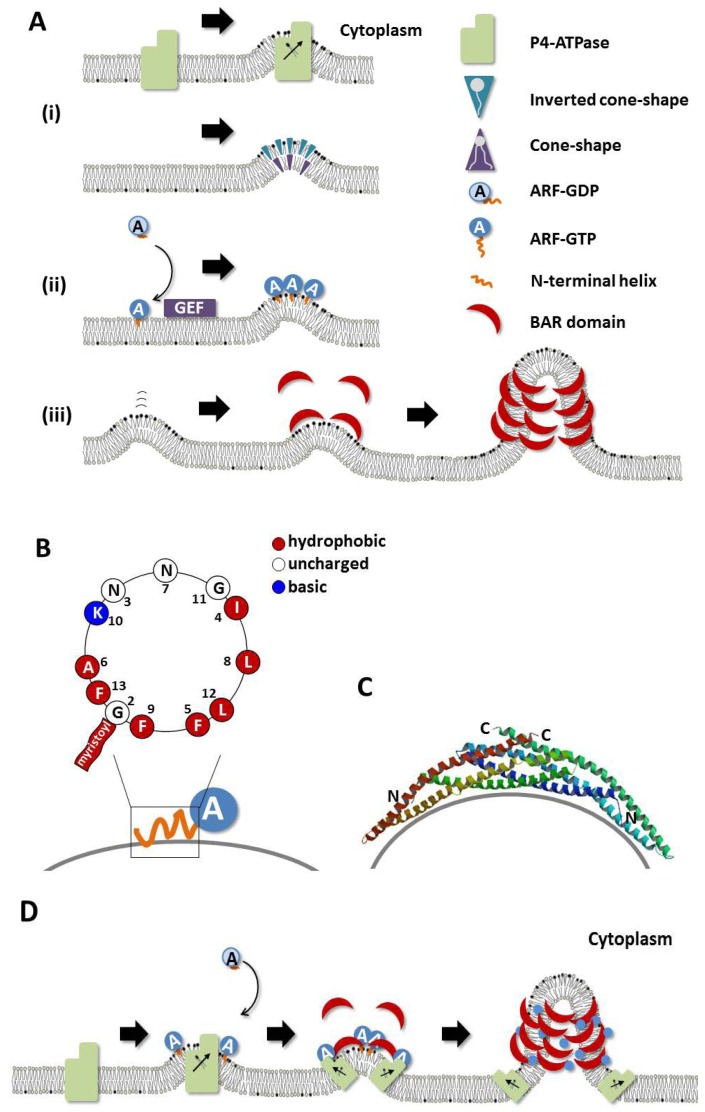
Mechanistic model for the generation of membrane curvature. (**A**) Membrane deforming mechanism (**i**) Lipid composition is changed by P4-ATPases, LPAT and PLA2; (**ii**) amphipathic helices of Arf family small GTPases are inserted into the cytoplasmic leaflet of the membrane; and (**iii**) the resultant curvature is sensed and stabilized by BAR domain proteins; (**B**) Helical-wheel representation of the N-terminal amphipathic helix of Arf1 (amino acids 2-13); (**C**) Structure of the BAR domain dimer of arfaptin. Image from the RCSB PDB [30] of PDB ID 1I49 [31]; (**D**) The complex regulation of proteins involved in generating membrane curvature.

Molecular biological approaches in yeasts and worm have revealed that the absence of specific P4-ATPases results in defects in membrane trafficking [14,15,18,21,22,23,24]. In the yeast *Saccharomyces cerevisiae*, five P4-ATPases are encoded in the genome (Table 1), and are involved in trafficking of proteins at different stages along the secretory and endocytic pathways [25]. For example, a *drs2*-ts mutant at the non-permissive temperature inhibits translocation of fluorescently labeled PS [26] and formation of post-Golgi vesicles [27], indicating that Drs2p is required to flip PS to the cytoplasmic leaflet of membranes in order to support vesicle formation from the *trans*-Golgi network (TGN). Alder-Baerens *et al.* [28] showed that loss of Drs2p and Dnf3p disrupts aminophospholipid translocation and phospholipid asymmetry in yeast post-Golgi secretory vesicles. In other organisms, including *Caenorhabditis elegans* and *Arabidopsis thaliana*, P4-ATPases also play essential roles in membrane trafficking [22,23,29]. For example, Poulsen *et al.* [29] showed that mutations of *ALA3* (a P4-ATPase in *A. thaliana*) result in impaired production of secretory vesicles from the Golgi. These findings confirm the critical roles of lipid flippases in vesicle formation. P4-ATPases may induce membrane bending via changing the distribution of phospholipids between the two leaflets of bilayers (Figure 1A).

**Table 1:** P4-ATpases in human, yeast, worm and plant.

Class	Human	*S. cerevisiae*	*C. elegans*	*A. thaliana*
1a	ATP8A1, ATP8A2	DRS2	TAT-1	ALA1
1b	ATP8B1, ATP8B2, ATP8B3, ATP8B4	-	TAT-2	ALA2-12
2	ATP9A, ATP9B	NEO1	TAT-5, TAT-6	
3	-	DNF1, DNF2	-	
4	-	DNF3	-	
5	ATP10A, ATP10B, ATP10D	-	TAT-3, TAT-4	
6	ATP11A, ATP11B, ATP11C	-	-	

Although there has been no direct evidence indicating that mammalian P4-ATPases also play essential roles in membrane trafficking, earlier studies showed that changes in lipid composition are involved in vesicular trafficking in a human erythroleukemia cell line, K562 [32]: endocytosis is stimulated by extracellular application of PS or PE, but partially inhibited by the addition of lyso-PS, which cannot translocate across the plasma membrane [32]. Thus, it is likely that the plasma membrane phospholipid asymmetry produces a driving force for budding of endocytic vesicles in mammalian cells as well.

Mammalian P4-ATPases have been implicated in various pathophysiologic processes. Among the 14 P4-ATPases (Table 1), mutations of ATP8B1 (FIC1) have been reported to cause familial intrahepatic cholestasis [33]. Furthermore, in ATP8B1-deficient mice, a significant amount of PS was observed in bile after infusion of bile salts, suggesting that ATP8B1 deficiency causes exposure of PS in the outer leaflet of the apical plasma membrane of bile canalicular cells [34]. The ATP8B3 gene is expressed in spermatocytes and spermatids [35]. In ATP8B3-null sperm cells, PS exposure was detectable before capacitation; the aberrantly early exposure of PS may affect the capacitation process [35]. The mouse ATP10A gene (also called ATP10C) is linked to diet-induced obesity and type II diabetes phenotypes [36], and an ATP10D mutation shows linkage to a fat-prone phenotype in certain strains of mice [37,38]. Recently, ATP11C has been shown to participate in the B lymphocyte differentiation [39,40]. Together, these observations imply that the asymmetric distribution of phospholipids in biological membranes maintained/regulated by P4-ATPases plays crucial roles in mammalian pathophysiology. However, the involvement of mammalian P4-ATPases in membrane trafficking, and the functional relationship between the P4-ATPase−related pathophysiology and membrane trafficking, remain poorly understood.

### 2.3. Lipid-Modifying Enzymes (LPAT and PLA_2_)

The size and chemical properties of different acyl chains and head groups of phospholipids can affect membrane curvature (Figure 1A) [41,42]. Phosphatidic acid (PA) and lyso-PA are interconverted by phospholipase A_2_ (PLA_2_) and lyso-PA acyl transferase (LPAT) activities, respectively. These lipids favor opposite membrane curvatures; lyso-PA and PA can induce budding towards the cytoplasm when located in the cytoplasmic and exoplasmic leaflets, respectively [43,44,45] (Figure 1A). Inhibitors of PLA_2_ prevent formation of membrane tubules from the Golgi apparatus and endosomes, and consequently inhibit protein trafficking through these organelles [44]. Conversely, application of specific LPAT inhibitors enhances tubulation of the Golgi [46]. Thus, local changes in the shape of phospholipids can influence membrane deformation and thereby the formation of carrier intermediates.

## 3. Proteins

### 3.1. Arf Family Small GTPases

Small GTPases act as molecular switches that regulate activity and localization of proteins and thereby determine the spatial and temporal organization of many cellular processes, including membrane trafficking. In their GTP-bound active state, members of the Arf family of small GTPases promote budding of coated carrier vesicles, such as those coated by the COPI complex and the clathrin/AP-1 complex [47]. Members of the Arf family (Arf1-Arf6) are related to a broad set of small GTPases [48]. These relatives include Sar1 and a subfamily of Arf-like (Arl) proteins. Arf1, the best-characterized member of the Arf subfamily, facilitates recruitment of COPI onto Golgi membranes and clathrin to late Golgi and endosomal compartments, both through its direct interaction with heterotetrameric adaptor complexes (AP-1, AP-3, AP-4) and monomeric adaptors (GGA1−GGA3) [49,50,51] and through activating lipid-modifying enzymes, including phospholipase D and phosphatidylinositol-specific kinases [52,53,54,55,56]. These data indicate that Arf1 acts as a master regulator of coated vesicle formation. 

Arf family proteins contain an N-terminal amphipathic helix and a myristoyl group that is usually attached to the N terminus (Figure 1A,B). Upon binding to GTP, the Arf molecule undergoes an extensive conformational change, during which the interswitch region displaces the N-terminal amphipathic helix from a hydrophobic pocket [57,58]. The exposed helix is in turn inserted into an adjacent lipid bilayer, and can induce membrane curvature (Figure 1A,B). Indeed, GTP-bound Arf and Sar1 molecules effectively tubulate liposomes, whereas GDP-bound forms do not [59,60,61,62,63,64]. In case of Sar1, the exposure of the amphipathic N-terminal helix triggers membrane curvature generation during budding of COPII-coated vesicles [60]. Mutations within the N-terminal amphipathic helix of Arf1 decrease the number of Arf1-induced tubules *in vivo* and its ability to tubulate liposomes *in vitro* [61,63]. Additional support of this idea is provided by the observation that many membrane-deforming proteins, such as endophilins [65,66], epsins [67] and amphiphysins [68,69], contain amphipathic helices that can be inserted into the lipid bilayers. These data support a mechanistic model in which insertion of N-terminal amphipathic helix of GTP-bound Arf proteins leads to bilayer bending upon vesicle budding (Figure 1A). 

### 3.2. BAR Domain-Containing Proteins

The BAR domain protein superfamily members have been implicated in membrane traffic, actin cytoskeleton remodeling and signal transduction [69,70,71]. BAR domains constitute a helical homodimer [69]. Given that BAR domain homodimers intrinsically adopt curved structures (Figure 1C), and that some BAR domain proteins, such as arfaptin and amphiphysin, can tubulate liposomes *in vitro* [68,69], it is likely that BAR domain proteins can generally sense and/or induce membrane curvature upon their recruitment from the cytosol onto the membrane surface [72,73,74]. Crystal structures, lipid-binding studies and liposome tubulation assays have validated the BAR domain−mediated membrane curvature model and expanded the superfamily; according to sequence and structural similarities, the family now comprises classical BAR domain proteins as well as N-BAR, F-BAR, I-BAR, BAR-PH, and PX-BAR proteins (see [73,75] for details). N-BAR domain proteins, such as amphiphysins and endophilins, have an N-terminal amphipathic helix, and may trigger membrane deformation via insertion into lipid bilayers. The BAR domain in turn senses curvature, and may thereby play roles in biogenesis of tubular organelles or the generation of endocytic vesicles or other carrier intermediates [65,69,76]. The observation of crystal structures and cryo-electron microscopic analyses suggest that BAR domains self-assemble into larger complexes, and that individual domains may be in contact in an organized manner and form BAR domain lattices [77,78,79,80]. F-BAR domain proteins, such as FCHo and FBP17, sense membrane curvature of clathrin-coated pits, oligomerize around the curved membranes, and ultimately induce invagination and tubulation of the plasma membrane during endocytosis [73,77,80,81]. BAR domains that sense, and presumably can generate curvature can be recruited or activated at buds, tubules or tubular-saccular structures during budding processes [74,82,83] (Figure 1A). 

In general, BAR domains bind to acidic membrane surfaces via basic residues on their concave face; the binding mode is relatively nonspecific, as compared with other membrane-binding modules that recognize head groups of specific phospholipids [69]. Many BAR domains are found in conjunction with other membrane-binding modules, such as PH (pleckstrin homology) and PX (phox homology) domains, which define membrane-binding specificity, and thereby functional specificity, of the BAR domain proteins [70]. For instance, PH domains, which recognize specific phosphoinositides, are required for correct membrane association of BAR domain proteins, APPL1 and ASAP1 (an ArfGAP) [84,85]. Moreover, a subset of the sorting nexin (SNX) family proteins, including SNX1 and SNX9, contain phosphoinositide-binding PX domains in addition to their BAR domains. The PX domain of SNX9 binds to phosphatidylinositol 4,5-bisphosphate (PtdIns(4,5)P_2_), and thereby assists in targeting of this SNX-BAR to PtdIns(4,5)P_2_-enriched regions of endocytic pits [86,87,88]. In contrast, the PX domain of SNX1 associates with the early and late endosomal phosphoinositides, respectively phosphatidylinositol 3-phosphate and phosphatidylinositol 3,5-bisphosphate, helping to target this protein to maturing early endosomes [89,90,91]. Therefore, the PX-BAR-containing SNXs can be selectively targeted to high-curvature membrane domains of cellular compartments that are enriched in specific phosphoinositides, and in turn drive membrane tubulation in the context of tubule-based sorting [92,93,94]. 

## 4. Interplay between Regulatory Proteins

### 4.1. Membrane Targeting of BAR Domain Proteins by Small GTPases

The BAR domain protein arfaptin was originally identified as a binding partner of the Arf family of small GTPases. We have recently shown that Arl1 (Arf-like 1) interacts with the BAR domain of arfaptin in a GTP-dependent manner [95]. Arl1 is localized primarily to the *trans* side of the Golgi complex, where it plays a role in membrane trafficking [96,97,98,99]. Overexpression of arfaptin induces Golgi-derived membrane tubules, whereas exogenous expression of Arl1 itself neither generates membrane tubules nor enhances arfaptin-mediated tubulation. However, association of arfaptin with Golgi membranes is abolished by depletion of endogenous Arl1 by RNAi, and rescued by exogenous Arl1 expression, indicating that Arl1 is required for targeting arfaptin to Golgi membranes. Although it is not clear whether association of Arl1 (or other proteins) or lipid modification provides the initial cue for membrane deformation, it is clear that arfaptin molecules recruited onto Golgi membranes through interaction with Arl1 sense and stabilize membrane curvature, and thereby facilitate tubule formation, probably through oligomerization like other BAR domain proteins [77,78,79,80] (Figure 1D). 

The early endosomal protein APPL1 interacts with Rab5 via its BAR and PH domains. This interaction is critical for targeting of APPL1 to endosomal membranes and APPL-mediated regulation of cell proliferation [85,100]. 

Thus, in addition to lipid-binding modules, such as PH and PX domains, the Arf and Rab families of small GTPases also determine the temporally and spatially confined localization of BAR domain proteins.

### 4.2. Arf Family Small GTPases and P4-ATPases

The first indication that P4-ATPases are involved in membrane trafficking came from an *Arf1* synthetic lethal screen in yeast, designed to discover proteins that function together with Arf in vesicle budding from the Golgi complex [15]. *DRS2* was identified in this screen. Drs2p localizes predominantly to the TGN; Drs2p-deficient cells are morphologically very similar to clathrin mutants [15]. As described above, Arf GTPases play an essential role in recruiting vesicle coat proteins, such as the COPI complex and the clathrin/AP-1 complex, from the cytosol, in order to induce their assembly on Golgi membranes. Genetic interactions were found between *drs2*, *arf* and clathrin mutant alleles, but not between *drs2* and COPI or COPII mutations. Consistent with these genetic interactions, clathrin-dependent pathways are strongly perturbed by loss of Drs2p, whereas COPI- and COPII-dependent protein trafficking pathways are unaffected [101,102]. Moreover, Drs2p has been shown to directly interact with Gea2p, a guanine nucleotide exchange factor for Arf GTPases [103]. Because the localizations of Gea2p, AP-1 and clathrin are not affected in *drs2* cells [102,103], it seems that Drs2p is not required for the recruitment of these proteins, but rather might be involved in the membrane deformation process by flipping phospholipids at sites of vesicle budding.

On the other hand, Neo1p, which is phylogenetically close to mammalian ATP9A and ATP9B (Table 1), exhibits genetic and physical interactions with Ysl2p, a potential guanine nucleotide exchange factor for Arl1p [104,105]. We and others have previously shown that Arl1 is localized to the TGN, where it regulates the retrograde trafficking from endosomes to the Golgi complex in mammalian cells [96,98,99]. Moreover, we have recently found that arfaptin (a BAR domain protein; see above) is a downstream effector of Arl1 [95], suggesting involvement of Arl1 in the membrane deformation machinery during vesicular trafficking (Figure 1D). Given that Neo1p may play a role in membrane trafficking within the endosomal/Golgi system [105], and that human ATP9A and ATP9B localize to the TGN and endosomes [106], ATP9A and ATP9B might be functionally implicated in the post-Golgi membrane trafficking (Figure 1B).

## 5. Conclusions

The curvature generation process is divided into two stages. The first stage is membrane deformation. The deformation can be induced by any of the following mechanisms: (i) changes in lipid composition (shapes, species and numbers of phospholipids) between two leaflets of the bilayer; (ii) insertion of small hydrophobic or amphipathic protein domains into one of the two leaflets; and (iii) binding of specific protein domains with an intrinsically curved shape (e.g., BAR domains) to the membrane surface, forcing the membrane to assume the shape of the protein domain. The second stage is curvature sensing, *i.e.*, the preferential recognition and stabilization of preexisting lipid topologies by protein domains with suitable curvature. We favor a model in which BAR domains function to sense and stabilize preexisting membrane curvature, rather than to induce curvature. However, the two functions are not mutually exclusive, and may occur simultaneously or in a complex fashion. Thus, generation of membrane curvature in the context of membrane trafficking and organelle dynamics is under complex regulation, involving changes in the composition and distribution of lipids, assembly of proteins, and the orchestrated activities of these regulatory proteins.
